# Crystal structure of a mirror-image L-RNA aptamer (Spiegelmer) in complex with the natural L-protein target CCL2

**DOI:** 10.1038/ncomms7923

**Published:** 2015-04-22

**Authors:** Dominik Oberthür, John Achenbach, Azat Gabdulkhakov, Klaus Buchner, Christian Maasch, Sven Falke, Dirk Rehders, Sven Klussmann, Christian Betzel

**Affiliations:** 1 Laboratory for Structural Biology of Infection and Inflammation, University of Hamburg, c/o DESY Building 22a, Notkestrasse 85, 22607 Hamburg, Germany; 2 Center for Free-Electron Laser Science, Deutsches Elektronen Synchrotron-DESY, Notkestrasse 85, 22607 Hamburg, Germany; 3 NOXXON Pharma AG, Max-Dohrn-Strasse 8-10, 10589 Berlin, Germany; 4 Institute of Protein Research, RAS, Pushchino, Moscow Region 142290, Russian Federation

## Abstract

We report the crystal structure of a 40mer mirror-image RNA oligonucleotide completely built from nucleotides of the non-natural L-chirality in complex with the pro-inflammatory chemokine L-CLL2 (monocyte chemoattractant protein-1), a natural protein composed of regular L-amino acids. The L-oligonucleotide is an L-aptamer (a Spiegelmer) identified to bind L-CCL2 with high affinity, thereby neutralizing the chemokine's activity. CCL2 plays a key role in attracting and positioning monocytes; its overexpression in several inflammatory diseases makes CCL2 an interesting pharmacological target. The PEGylated form of the L-aptamer, NOX-E36 (emapticap pegol), already showed promising efficacy in clinical Phase II studies conducted in diabetic nephropathy patients. The structure of the L-oligonucleotide·L-protein complex was solved and refined to 2.05 Å. It unveils the L-aptamer's intramolecular contacts and permits a detailed analysis of its structure–function relationship. Furthermore, the analysis of the intermolecular drug–target interactions reveals insight into the selectivity of the L-aptamer for certain related chemokines.

## Abstract (web summary)

Spiegelmers are ‘mirror image' L-ribose oligonucleotides being developed as therapeutics. Here the authors present a crystal structure of the therapeutic L-aptamer NOX-E36 bound to the pro-inflammatory chemokine CLL2, providing insight into NOX-E36's selectivity and mode of action.

## Graphical abstract

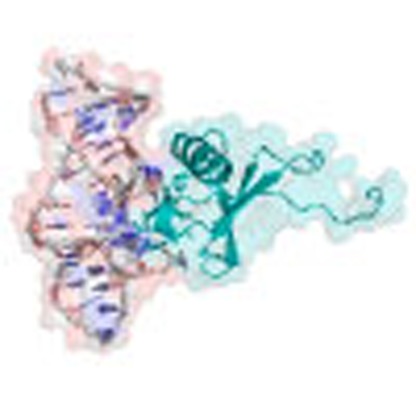

CCL2 (Monocyte Chemoattractant Protein-1; MCP-1) is a member of the CC-chemokine family, a group of small secreted proteins of 8–10 kDa that regulate leukocyte migration in the human body^1^. CCL2 acts as a strong chemoattractant primarily for monocytes, but also for memory T cells and natural killer cells^1^.

Whereas other chemokines are able to bind and activate several members of the chemokine receptor family, which all belong to the group of G protein-coupled receptors, CCL2 binds with high affinity almost exclusively to chemokine receptor 2 (CCR2)^2^. On the other hand, CCR2 can be activated also by other monocyte chemoattractant proteins that are closely related to CCL2, that is, CCL8 (MCP-2), CCL7 (MCP-3) and CCL13 (MCP-4)^2^.

Similar to other chemokines, CCL2 can form dimers and higher oligomers^3^. Whether CCL2 can bind to the receptor as a dimer with reasonable affinity is still a matter of debate. Following a widely accepted model, receptor binding of CCL2 and chemokines in general takes place in a two-step process: the core domain of the chemokine binds to the N-terminus of the receptor followed by an interaction of the N-terminus of the chemokine with the helical bundle of the receptor^3^. Since in the case of CCL2 N-terminal residues are also involved in dimer formation, the second step leading to receptor activation very likely requires the monomeric form. Yet, dimerization and glucosaminoglycan (GAG)-binding are important for biological activity *in vivo*^4^. According to the currently accepted view, monocytes encounter CCL2 bound to GAGs on the surface of endothelial cells, which finally leads to cell arrest and transmigration. In inflamed tissue, monocytes continue to migrate along the CCL2 gradient produced by macrophages^5^. By attracting and activating immune cells, CCL2 plays a pivotal role in many inflammatory processes and has been shown to be involved in a wide variety of diseases with or without an obvious inflammatory aspect as, for example, rheumatoid arthritis, systemic lupus erythematosus, multiple sclerosis, atherosclerosis, allergy and asthma, diabetic retinopathy, lupus nephritis, diabetic nephropathy and others^5^,^6^,^7^.

For therapeutic interventions, small-molecule CCR2 inhibitors and antibodies against CCR2 or CCL2 (ref. 5) have been developed to interfere with CCL2—CCR2 signalling. However, none of these pharmacological modalities have reached the market so far. In an alternative approach to block the activity of CCL2, we generated a CCL2-binding mirror-image aptamer consisting of L-ribonucleotides, a so-called Spiegelmer^8^.

Spiegelmers are chemically synthesized L-stereoisomer oligonucleotide aptamers, which are biologically very stable and immunologically passive because of their non-natural mirror-image conformation^9^. In order to identify a Spiegelmer, first aptamers are selected from oligonucleotide libraries to bind to the non-natural, mirror-image form of an intended target molecule (in this case mirror image or D-CCL2) by an evolutionary screening technique called SELEX^10^. By chemical synthesis of the selected aptamer sequence from non-natural L-nucleotides, an exact mirror image of the aptamer is produced, the Spiegelmer, which consequently binds to the natural L-protein (in this case L-CCL2). For *in vivo* applications, Spiegelmers are often conjugated to a 40-kDa polyethylene glycol to retard their renal elimination, thus improving their pharmacokinetic characteristics.

A mouse-CCL2-specific Spiegelmer (mNOX-E36) was shown to be active in several animal models^11^,^12^,^13^ and its human-specific counterpart NOX-E36 (emapticap pegol) has already been tested successfully in a Phase IIa study in diabetic nephropathy patients^14^.

To gain a better understanding and to obtain structural details of how natural protein targets in the L-configuration are bound by non-natural oligonucleotides in the L-configuration, we crystallized the L-protein·L-oligonucleotide complex composed of L-CCL2 and the L-oligonucleotide part of NOX-E36. The structure was solved applying single-wavelength anomalous diffraction (SAD) and refined to 2.05 Å. Together with the structures reported in the accompanying paper from ref. 15, these are the first three-dimensional (3D) structures of L-oligonucleotide aptamers in complex with their natural L-protein targets.

## Results

### General description of the crystal

Purified human CCL2 was complexed with the oligonucleotide part of NOX-E36, that is, the molecule without the PEG moiety. Because no suitable search model of the complex was available for molecular replacement calculations, selenium was introduced at L-U31 of the oligonucleotide to enable SAD phasing. Introduction of selenium was shown earlier to facilitate structural analysis of natural oligonucleotides^16^ and allowed to solve the phasing problem also in this case. Crystals of the complex were obtained by vapour diffusion and diffraction data up to 2.05 Å were collected at the PETRA III beamline P13. Initial phases could be obtained applying experimental SAD phasing^17^, followed by density modification and subsequent iterative refinement and model-building cycles. Crystal parameters and refinement statistics are summarized in Table 1.

**Figure 1:**
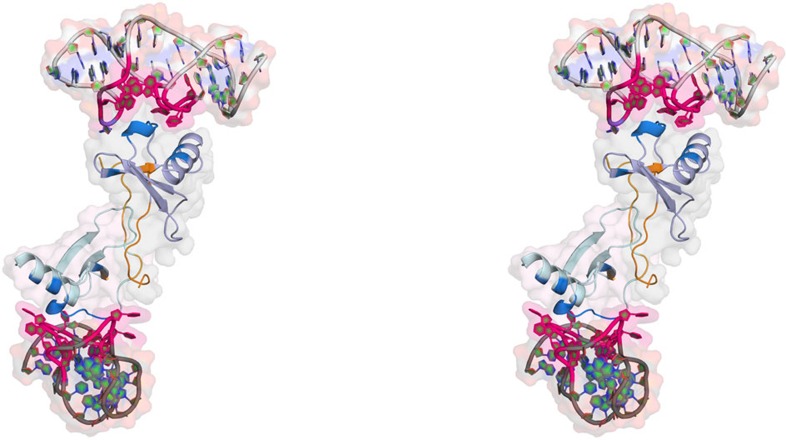
Stereo figure of the NOX-E36 L-aptamer·CCL2 complex. The structure is shown as the biological assembly (dimer) at 2.05 Å resolution. One monomer of the complex is present in the asymmetric unit. The L-aptamer-backbone is shown in light and dark grey, the binding region to CCL2 is highlighted in magenta. CCL2 is coloured in purple and light blue, residues binding directly to the L-aptamer are displayed in dark blue, and residues facilitating dimerization through intermolecular hydrogen bonds are presented in orange.

In the asymmetric unit of the tetragonal space group, one complex of one protein molecule bound to one L-oligonucleotide molecule is present. An analysis of all contacts including symmetry-related molecules using the programmes Coot and PISA clearly shows that the complex is a stable dimer of two CCL2·L-aptamer complexes, which are connected through interaction of two CCL2 molecules (Fig. 1). The first three N-terminal residues of CCL2 in the complex were disordered and thus omitted from the model.

### Comparison of native and L-aptamer-bound CCL2 structures

The overall 3D structure of CCL2 bound to the L-oligonucleotide is very similar to native uncomplexed CCL2 (ref. 18; PDBs 1DOL and 1DOK; Supplementary Fig. 1a) with an overall root mean square deviation (r.m.s.d.) of 0.60 Å (superposition with PDB 1DOL) or 0.41 Å (superposition with PDB 1DOK). For comparison, superposition of these two uncomplexed structures yields an r.m.s.d. of 0.50 Å (see Supplementary Table 1 for more details). No significant deviations of *C*_α_ can be observed; thus, the overall 3D structure of the protein is not altered on binding to the L-aptamer. As expected, deviating orientations of flexible amino-acid side chains are observed in the binding region, especially concerning Arg18, Lys19, Arg24 and His66. Notably, a comparison of the uncomplexed CCL2 structures also shows significantly deviating side chain orientations. Only Lys19 in our structure shows a unique orientation, whereas all other amino-acid side chains in the binding region adapt a conformation that is highly similar either to the one observed in PDB 1DOL or 1DOK (Supplementary Fig. 1a,b). As in our structure, CCL2 was present as a stable dimer in the crystal (PDB: 1DOL and 1DOK)^18^ and NMR (nuclear magnetic resonance; PDB: 1DOM)^19^ structures of native CCL2. Aligning whole dimers (instead of the monomers) by their dimerization sites shows that the dimerization sites themselves almost perfectly superimpose; however, the orientation of the monomers towards each other in the respective structures differs slightly, which has been reported already^18^ for a comparison of PDBs 1DOL, 1DOK and 1DOM. As a consequence, considerable differences are seen from residue 19 onwards (Supplementary Fig. 1c).

### Structure of the NOX-E36 L-aptamer

**Figure 2:**
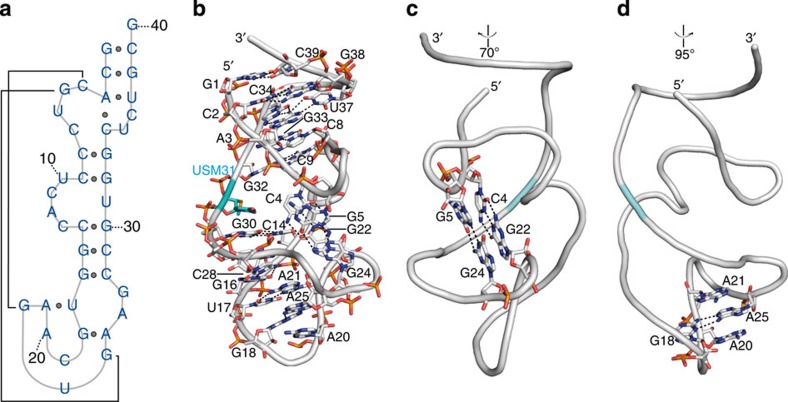
Structure of the NOX-E36 L-aptamer. (**a**) Secondary structure of the NOX-E36 L-aptamer derived from the three-dimensional (3D) structure. (**b**) Representation of the main intramolecular interactions of the L-oligonucleotide. The seleno-modified uridine (U31) on the left side of the L-aptamer (highlighted in cyan) is located opposite to the target-binding pocket (G5-C11 and G22-G27). (**c**) The pseudoknot-motif involving the base pairs C4-G22 (Watson–Crick) and G5-G24 (noncanonical) is highlighted. Turned with respect to **b** as indicated. (**d**) A25 is flipped out of the helix and stabilized in this position through stacking between A21 and A20 and the noncanonical base pairing with G18. Turned with respect to **b** as indicated.

The NOX-E36 L-aptamer forms a heavily distorted hairpin structure, whose exterior shell is rod-shaped with a length of 4.5 nm and a diameter of 2.5 nm (Fig. 2b). An analysis of the *χ* torsion angles shows that most residues are in *anti* conformation, whereas residues G5, C7, A21, A25 and G27 are in *syn* conformation. All of the latter nucleotides are either directly involved in CCL2 binding (G5, C7 and G27) or located close to the protein·L-aptamer interface (A21 and A25). In regard of sugar-puckering, A21 is in C1′-exo state, G27 is in C4′-exo state, whereas all other nucleotides are found in either of the energetically preferred states C3′-endo (1–4, 8,9, 12–20, 24, 28–33 and 35–40; in total 28 of the 40 residues) or C2′-endo (5–7, 10, 11, 22, 23, 25, 26 and 34).

The secondary structure of the L-aptamer (Fig. 2a) is quite different from the secondary structure predicted for the uncomplexed L-aptamer, computed using the software mfold^20^ (Supplementary Fig. 2). In total, the structure comprises nine Watson–Crick, two Hoogsteen and two noncanonical base pairs, as summarized in Supplementary Table 2 and also highlighted in Fig. 2b. The left-handed (counterclockwise, 5′–>3′), terminal helix is a noteworthy feature, as natural D-RNAs have right-handed (clockwise, 5′–>3′) helices. Remarkably, the loops forming the target-binding site are interconnected by two base pairs in parallel strand orientation (C4-G22 and the noncanonical GG N7-N1 carbonyl-amino base pair G5-G24), forming a mini pseudoknot (Fig. 2c). Another interesting intramolecular interaction feature involves nucleotide A25, which is flipped out of the helix, stacks with A20 and A21, and forms a noncanonical GA N3-amino amino-N1 base pair with nucleotide G18 (Fig. 2d). These interactions are essential structural features, defining and stabilizing the shape of the target-binding site.

After careful investigation of the mFo–dFc difference electron density maps and anomalous difference maps calculated from diffraction data collected at 0.978 Å wavelength, a total of seven ion positions could be identified. At one position, the relatively strong anomalous signal (12.7*σ*, strongest peak after that at the position of Se, see Supplementary Fig. 3a,b) and the 2mFo–mFc and mFo–dFc electron densities indicated the presence of an ion heavier than K^+^ or Ca^2+^. Considering the buffer composition and crystallization conditions, one Sr^2+^ was positioned here. Rb^+^ would show similar electron density as well as anomalous scattering at 0.978 Å wavelength; however, the resulting ion–oxygen distances (on average 2.47±0.09 Å) corrrespond much closer to Sr-O distances reported in the literature^21^ (2.62 Å) than to that reported for Rb-O^22^ (2.98 Å). Removal of the ion, performing simulated annealing refinement to avoid possible model bias and introducing Sr^2+^ or Rb^+^ back and performing refinement with manually added tight geometrical restraints on the ion–oxygen distances resulted in ion–oxygen distances of 2.55±0.08 Å for Sr-O and 2.64±0.20 Å for Rb-O, indicating a much better agreement with the ideal Sr-O distances than the Rb-O distances. At five of the other six identified ion positions, anomalous difference density peaks could be found extending to 7.28, 7.16, 5.74, 5.29 and 4.91*σ*, respectively. At one ion position, no peak in the anomalous difference density map was observed (see Supplementary Fig. 3c–e). Tentatively, K^+^ ions were positioned in the former cases and Na^+^ in the latter case, followed by ion identification as described for Sr^2+^ (see Supplementary Table 3). A detailed analysis of the resulting models with Coot^23^ and applying the CheckMyMetal-server^24^ confirmed that the ion-binding sites were correctly assigned. Three of the potassium ions and the sodium ion each bind to one nucleotide residue. One potassium ion (K4) binds to two neighbouring aptamer residues (to O6 of G32 and G33) and one potassium ion (K3) is bound to the aptamer indirectly through interactions within the water network. Two of the potassium ions (K1 and K2) are bound to symmetry-related aptamer chains and are thus stabilizing the crystal lattice. Both ions, K4 and Na^+^, are located within an extended water network that interconnects the 3′- and the 5′-end of the aptamer (see Supplementary Figs 3c,d and 4). The bivalent ion Sr^2+^ interconnects three residues (C9, C11 and A12) belonging to the binding region of the L-aptamer (Supplementary Fig. 5). Two of these, C9 and C11, form hydrogen bonds with CCL2. In addition, Sr^2+^ is coordinated by three water oxygens and is essential for an extended water network that further stabilizes the binding pocket. Sr^2+^ is the most abundant bivalent ion in the crystallization buffer; we expected that either Ca^2+^ or Mg^2+^ would take this position under physiological conditions. We investigated the ion dependence of L-aptamer binding using surface plasmon resonance (SPR), revealing a strong dependence on Ca^2+^, which appears to be important for proper folding of the L-aptamer and will likely occupy the position of Sr^2+^ observed in the structure. In contrast, Mg^2+^ had only a minor effect (Supplementary Fig. 6). Up to a physiological concentration, Na^+^ accelerates complex formation, but Na^+^ concentrations above physiological levels lead to reduced binding. K^+^ did not show any effect on the binding kinetics.

As mentioned before, this structure was solved using the Se-SAD phasing. For this purpose, a Se-labelled L-aptamer was synthesized by replacing uridine at position 31 (U31, highlighted in Fig. 2b) with 2′-methylseleno-uridine. This modification is located at the surface and apparently does not change the overall structure. Since the seleno-modified residue is located opposite to the region interacting with CCL2, it also does not influence or interfere with target-binding.

### Interactions between L-aptamer and L-CCL2

**Figure 3:**
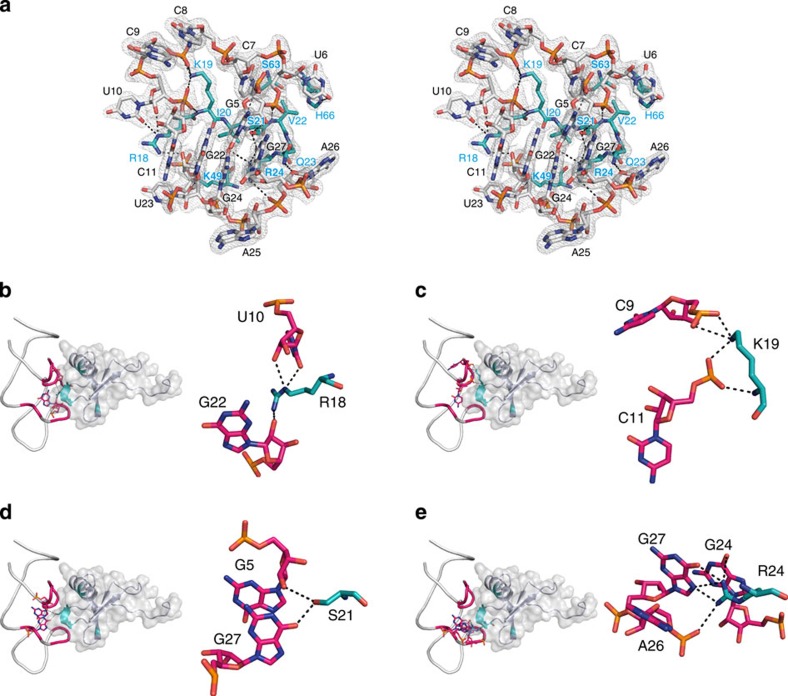
Interactions between L-aptamer and CCL2. (**a**) Stereo image showing a detailed view of the interactions between the L-aptamer's binding pocket (nucleotides G5-C11 and G22-G27; grey) and the CCL2 epitope (amino acids 18–24, 49, 63 and 66; cyan). Only residues are depicted that are directly involved in binding via hydrogen bonds (shown as dashed lines). The model is superimposed to the final 2Fo–Fc electron density map contoured at 1.0 *σ*. (**b**–**e**) Close-ups of selected L-aptamer·CCL2 interactions in which CCL2 residues are highlighted in blue and the L-aptamer residues are shown in magenta; on the left side of each close-up an overview of the complex is depicted.

The L-aptamer's target-binding site is a pocket shaped by nucleotides 5–11 (upper part of the pocket as shown in Fig. 3a) and nucleotides 22–27 (lower part in the figure). A protruding patch of mostly basic and polar amino acids of CCL2, that is, amino acids 17–24, inserts into this pocket. Each amino acid within this stretch, besides Asn17, is interacting directly with the L-aptamer via at least one hydrogen bond or electrostatic interaction. In addition, the upper part of the pocket forms interactions with Ser63 and His66 of the CCL2 α-helix and Lys49 interacts through one hydrogen bond with U23 of the lower part of the binding pocket. In total, 10 amino-acid and 11 nucleotide residues are involved in binding mediated through hydrogen bonds, electrostatic interactions and at least one cation–*π* interaction. The L-RNA·protein interface comprises 17 nucleotides and 17 amino acids with a calculated interface area of 714.3 Å^2^ (protein: 748.2 Å^2^, RNA: 680.4 Å^2^; analysed using PISA^25^), corresponding to ∼14% of the solvent-accessible area of CCL2.

A few examples showing the complexity of the interactions are highlighted in Fig. 3b–e and described in the following: amino acid Arg18 forms two hydrogen bonds between the δ-nitrogen and both 2′-OH and O2 of U10 as well as one hydrogen bond between the Ω-nitrogen and the 2′-OH of G22 (Fig. 3b). Lys19 forms four polar contacts (hydrogen bonds and electrostatic interactions) with the phosphate oxygens of nucleotides C9 and C11 (Fig. 3c). The side chain hydroxyl group of Ser21 makes hydrogen bonds with the G5 2′-OH and the G27 O6 (Fig. 3d). The Arg24 Ω-nitrogens bind to the nucleobases of both G24 and G27 and to a phosphate oxygen of A26; furthermore, there is a cation–*π* interaction between the side chain and the G24 nucleobase (Fig. 3e). Details for further interactions involving Ile20, Val22, Gln23, Lys49, Ser63 and His66 are shown in Supplementary Fig. 7.

The NOX-E36 nucleotide sequence appears to be quite sensitive to mutations. This is supported by the fact that after *in vitro* selection, the identified clones mainly consisted of only one sequence from which NOX-E36 was derived by truncation of primer-binding sites. Only a few sequences differed by one or two point mutations^8^. In these rare cases, different nucleotides were observed at positions 5, 19, 29, 31, 32, 36 and 40. Of these nucleotides, only G5 in NOX-E36 is involved in target binding and three are engaged in base pairing (positions 5, 29 and 32) as is now evident from the structure. All but one of the mutated molecules were inactive. Only the molecule showing a U31C mutation displayed an unaltered affinity and, therefore, the seleno-modified uridine was introduced at this position.

### Interference with CCL2 dimerization

**Figure 4:**
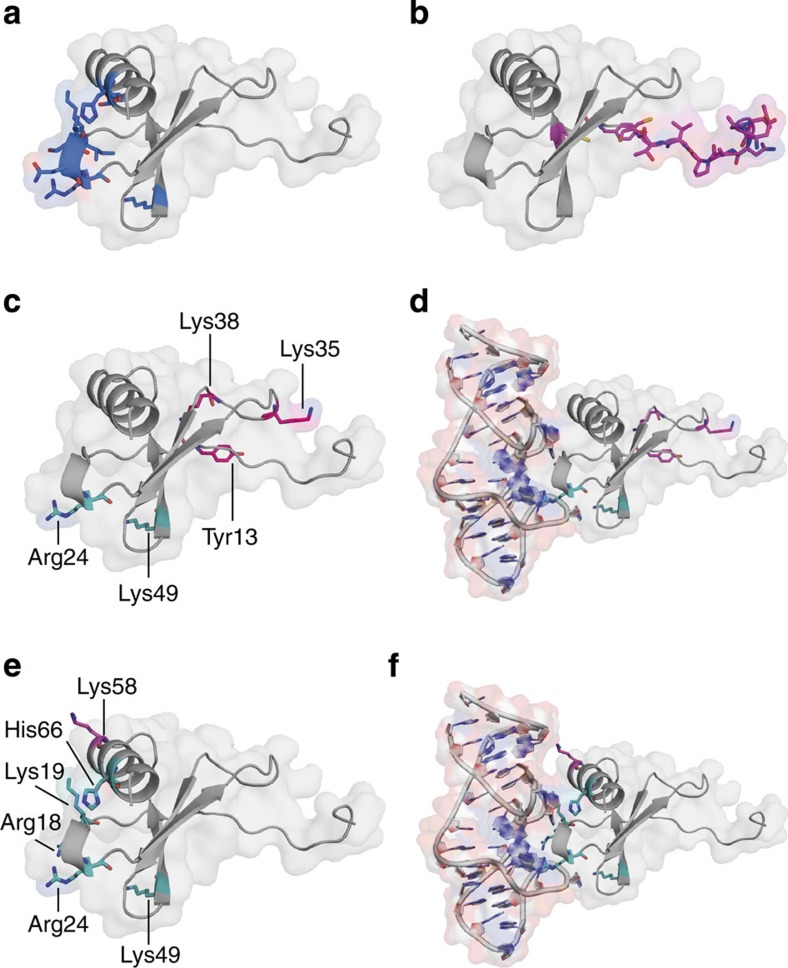
Oligomerization, receptor-binding and GAG-binding region of CCL2. (**a**) Detailed view of the L-aptamer-binding region of CCL2. Amino acids involved in binding are highlighted in blue and shown as sticks. (**b**) Dimerization region of CCL2. The dimerization region (coloured amino acids) is located opposite to the L-aptamer-binding region. (**c**) Receptor binding of CCL2. Residues involved in both receptor and L-aptamer binding are depicted in cyan, whereas those only involved in receptor-binding are shown in magenta. (**d**) Same as **c** but with the L-aptamer added. (**e**) GAG binding of CCL2. Residues involved in both GAG and L-aptamer binding are highlighted in cyan, whereas the one residue involved only in GAG binding is shown in magenta. (**f**) Same as **e**, but with the L-aptamer that shields almost the complete GAG-binding site.

As mentioned before, CCL2 dimerization is essential for its function *in vivo*^4^. The total dimer interface area, formed by 21 of the 66 residues of each CCL2 monomer, is 809.2 Å^2^. The CCL2 dimer is stabilized by 10 hydrogen bonds involving residues Ile5, Asn6, Val9, Thr10, Cys11, Tyr13, Asn14 and Cys52 (highlighted in orange in Fig. 1). Cys52 is linked to the N-terminal region by an intramolecular disulphide bond with Cys12, and its main chain nitrogen forms a hydrogen bond to Asn6 of the other chain of the dimer. These residues are located opposite to the epitope recognized by the L-aptamer (Figs 1 and 4a,b). Interference of NOX-E36 with oligomerization can thus be excluded and *vice versa*, CCL2 dimerization does not impair binding of the L-aptamer.

### Interference with CCL2 receptor and GAG binding

Binding of CCL2 to its receptor CCR2 is mediated by two clusters of primarily basic residues (Arg24, Lys35, Lys38, Lys49 and Tyr13), separated by a hydrophobic groove^26^. Furthermore, Tyr13 together with the N-terminal domain is also important for triggering signalling through CCR2 (ref. 27; Fig. 4c). The L-aptamer binds directly to Arg24 and Lys49 and thus shields an essential part of the CCL2 receptor-binding region, blocking CCL2's interaction with its receptor CCR2 (Fig. 4c,d).

Heparin-binding studies indicated that amino acids Arg18, Lys19, Arg24, Lys49, Lys58 and His66 are important residues mediating CCL2 binding to GAG^28^,^29^ (Fig. 4e), which is essential for the formation of a chemotactic gradient and thus for CCL2's *in vivo* function^4^. Since the L-oligonucleotide forms at least three hydrogen bonds with each of the amino acids Arg18, Lys19 and Arg24 and one hydrogen bond with His66 and Lys49, respectively, almost the complete GAG-binding region of CCL2 is covered by the L-aptamer (Fig. 4e,f). It can be assumed that blocking both receptor and GAG-binding regions contributes to the strong *in vivo* effects of the NOX-E36 L-aptamer.

### Binding of the NOX-E36 L-aptamer to related chemokines

**Figure 5:**
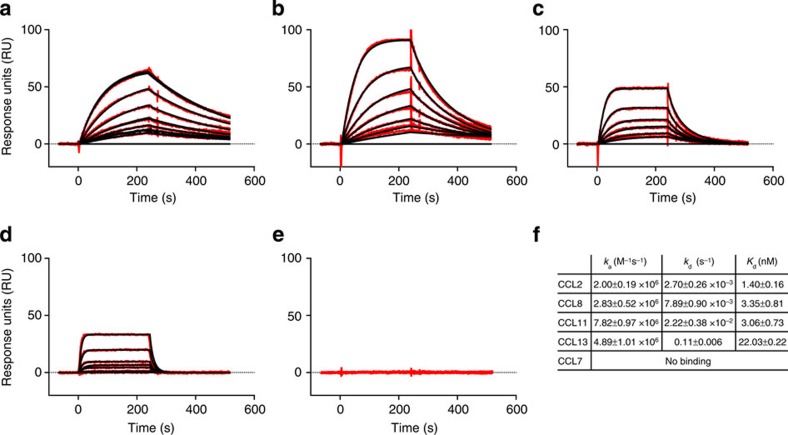
NOX-E36 L-oligonucleotide binding to CCL2 and related chemokines. Sensorgrams and kinetic binding parameters of the NOX-E36 L-aptamer binding to immobilized human CCL2/MCP-1 (**a**), CCL8/MCP-2 (**b**), CCL11/Eotaxin (**c**), CCL13/MCP-4 (**d**) and CCL7/MCP-3 (**e**) as determined by surface plasmon resonance measurements (Biacore). Red: raw data, black 1:1 Langmuir fitting. The dissociation constants *K*_d_ and their kinetic rate constants *k*_a_ and *k*_d_ (**f**) were analysed under physiological buffer conditions at 37 °C. Numerical values are means±s.e.m. The mean *K*_d_s were calculated from individually determined *K*_d_s and not from mean *k*_a_ and *k*_d_. The *K*_d_ for CCL8/MCP-2 (*n*=5) is twofold increased compared with CCL2/MCP-1 (*n*=7) with increased association (*k*_a_) and dissociation rate constants (*k*_d_), indicating a reduced number of contact points. For binding to CCL11/Eotaxin (*n*=4), a more pronounced loss of complex stability (that is, faster dissociation rate constant) as compared with CCL8 was observed, whereas the target association rate constant is further increased. The L-aptamer binding to CCL13/MCP 4 showed a clearly reduced affinity (*n*=3). No binding of CCL7 was observed (*n*=3).

**Figure 6:**
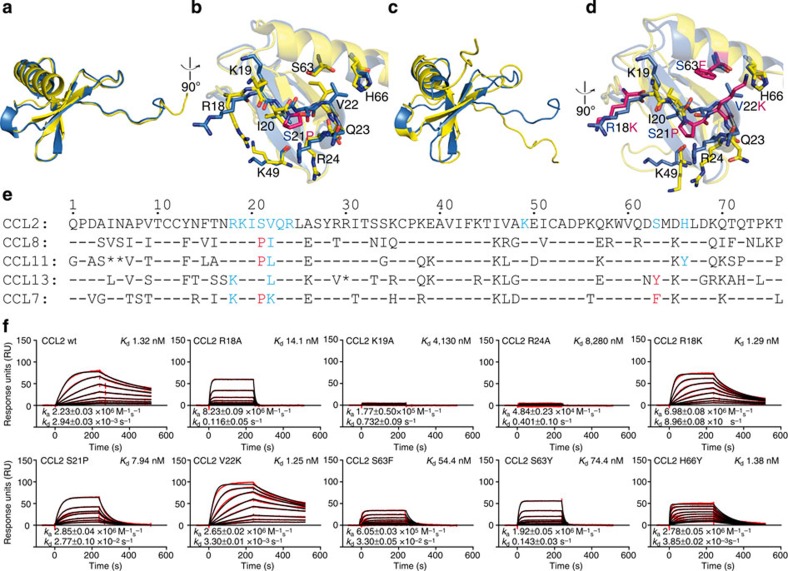
Overall structure and L-aptamer-binding site of CCL2 and related chemokines. (**a**) Comparison of CCL2 (blue) with CCL8 (yellow, PDB 1ESR (ref. 27)). The overview of the aligned monomers shows a virtually identical topology. (**b**) Comparison of the L-aptamer-binding site of CCL2 with the corresponding region of CCL8. Residues involved in binding are shown in dark blue for CCL2 and yellow for CCL8. The proline in the epitope of CCL8 that is present instead of serine at position 21, is highlighted in magenta. (**c**) Comparison of CCL2 (blue) with CCL7 (yellow, PDB 1BO0 (ref. 30)). The overview of the aligned monomers shows a very similar topology. (**d**) Comparison of the L-aptamer-binding site of CCL2 with the corresponding region of CCL7. Residues involved in binding are shown in dark blue for CCL2 and yellow for CCL7. The residues in the epitope of CCL7 that are different from those in CCL2 are highlighted in magenta. (**e**) Sequence alignment, the epitope in CCL2 as well as sequence deviations neutral to the binding are highlighted in cyan; sequence deviations with a negative effect on NOX-E36 binding are highlighted in red. Sequence identity is indicated by a dash, empty positions are marked with an asterisk. (**f**) Surface plasmon resonance measurements of L-aptamer binding to wild-type CCL2 and single point mutants.

Binding experiments show that the NOX-E36 L-aptamer not only recognizes CCL2/MCP-1 but also the closely related chemokines CCL8/MCP-2, CCL11/eotaxin and CCL13/MCP-4 with moderately reduced affinities; however, CCL7/MCP-3 is not bound (Fig. 5). To investigate whether these differences can be explained by structural features, we aligned available structures of these chemokines (PDBs 1ESR^30^ (CCL8), 1EOT^31^ (CCL11), 2RA4 (ref. 32; CCL13), 1BO0 (ref. 33; CCL7)) with the structure of the L-aptamer·CCL2 complex. We found a high degree of similarity concerning the overall structures and importantly also concerning the backbones of the amino acids that comprise the corresponding binding epitope; differences are mostly restricted to conformations of flexible side chains (Fig. 6a–d). The r.m.s.d. values obtained after superposition of all atoms are (relative to CCL2 in complex with NOX-E36) 0.67 Å for CCL8 and 0.66 Å for CCL13. For CCL11 and CCL7, only structural models derived from NMR experiments are available and the r.m.s.d. values are slightly larger: 1.22 Å for CCL11 and 1.46 Å for CCL7. Since different side chain conformations were observed also in different structures of CCL2 as discussed above, we expect that the side chains of CCL2-related chemokines could rotate into a position compatible for binding as observed in our structure. However, the chemokines show a few conspicuous sequence deviations within the epitope, which might account for the altered affinities of the L-aptamer (Fig. 6e). In CCL2, both the δ- and an Ω-nitrogen of the Arg18 side chain are engaged in interactions with the L-aptamer (Fig. 3b), whereas CCL13 and CCL7 have a lysine at this position. We assumed that a lysine might make fewer contacts, with the result of a reduced affinity. The side chain hydroxyl group of Ser21 in CCL2 forms two hydrogen bonds with the L-aptamer (Fig. 3d), whereas CCL8, CCL11 and CCL7 show a proline in this position, which cannot make hydrogen bonds (Fig. 6a–e). CCL2 residue Val22 is bound by its main chain nitrogen (Supplementary Fig. 7b). CCL8, CCL11 and CCL13 show very conservative exchanges in this position (Ile or Leu), which sterically appear to be fully compatible with L-aptamer binding. In contrast, CCL7 has a lysine in this position, which might cause steric hindrance of binding. The same functional group of the L-aptamer that binds to Ile20, that is, C7 O4, also binds to the side chain of Ser63 (Supplementary Fig. 7a). In CCL13 and CCL7, Ser63 is replaced by a bulky aromatic amino acid (Tyr or Phe, respectively), which clashes with nucleotide C7 in our alignment. Besides the loss of the interaction with Ser63, the interaction between C7 and Ile20 might also be affected by conformational rearrangements necessary to avoid a clash. Finally, the τ-nitrogen of CCL2 residue His66 binds to the O4 of nucleotide U6 (Supplementary Fig. 7b); CCL11 has a tyrosine in this position. In this case, we expected that the *p*-hydroxyl group of CCL11's Tyr66 could enter the same interaction. Notably, all sequence deviations suspected to weaken the interaction with the L-aptamer are combined in CCL7 (Fig. 6d,e), which is not bound.

To underpin these considerations with functional data, we produced wild-type CCL2 and single-point mutants corresponding to the mentioned sequence deviations, that is, R18K, S21P, V22K, S63F, S63Y and H66Y, and studied the effect of the mutations on L-aptamer affinity using SPR (Fig. 6f). For comparison, we also tested mutants R18A, K19A and R24A, which were made with the intent to disrupt the strongest interactions seen in our structure (see Fig. 3). The correct folding of the recombinantly expressed native CCL2 and CCL2 mutants was established by testing their ability to activate the CCR2 receptor in a cell-based chemotaxis assay (Supplementary Fig. 8). Only the R24A mutant showed a drastically reduced receptor activation, which had to be expected: this mutant is known to show a strongly reduced affinity for the receptor^27^.

SPR measurements revealed that the L-aptamer's affinity for the CCL2-mutant R18A is about 10-fold reduced (*K*_d_ 14.1 nM; wild type: 1.32 nM), whereas the K19A and R24A mutations entailed a much more pronounced loss of binding affinity (*K*_d_ 4.13 and 8.28 μM, respectively). This confirms that amino acids K19 and R24, which are fully conserved among the CCLs considered here, are essential interaction partners for the L-aptamer.

The mutations R18K, V22K and H66Y had no negative effect on the dissociation constant *K*_d_ and the kinetic rate constants *k*_a_ and *k*_d_ for V22K and H66Y are very similar to those observed for the wild type, indicating that Lys22 does not cause steric constraints and Tyr66 can indeed contribute to the binding. In case of R18K, both the on-rate and the off-rate were about threefold slower compared with the wild type. In contrast, a reduced affinity of the L-aptamer for all other mutants was observed. The dissociation constants determined for mutants S21P, S63F and S63Y were 7.94, 54.4 and 74.4 nM, respectively.

## Discussion

We describe the first high-resolution crystal structure of a non-natural, mirror-image L-RNA aptamer binding to a natural L-protein. The L-protein is CCL2, a chemokine involved in inflammatory processes that are dysregulated in several diseases^7^. The PEGylated form of the L-aptamer (Emapticap pegol) has already been demonstrated to be safe and well tolerated in several Phase I studies in healthy volunteers^34^. Recently, this substance also showed efficacy in diabetic nephropathy patients in a Phase IIa clinical study^14^. Since this is the first mirror-image oligonucleotide that was developed into clinical studies, we sought to understand more about the molecular details of the target recognition of an L-aptamer that finally builds the basis for its efficacy.

The first idea about potential interactions within an RNA oligonucleotide is usually yielded by computing a secondary structure model under the assumption of a minimum free energy. It is striking that the secondary structure of the CCL2-bound L-oligonucleotide derived from the crystal structure (Fig. 2a) is completely different from the secondary structure computed by the RNA secondary structure prediction software mfold^20^ for the free oligonucleotide (Supplementary Fig. 2). It is not unusual that the aptamer structure predicted as the most stable one is not the structure finally adopted by the molecule^35^. The complexity of the NOX-E36 L-oligonucleotide structure, especially concerning the parallel-stranded pseudoknot motif around base pairs C4-G22 and G5-G24, the non-canonical base pairings between G5-G24 and G18-A25 and the intercalation of A25 between A20 and A21, is currently beyond computational predictability.

The NOX-E36 L-oligonucleotide binds to its target at a surface region with positive electrostatic potential, which is a commonly described characteristic of aptamer binding^35^. In total, we found 19 direct hydrogen bonds and one electrostatic interaction between the binding partners. Furthermore, we identified one cation–*π* interaction (Arg24—G24), which is known to be an important stabilizing structural feature for protein–nucleic acid complexes^36^,^37^,^38^. The observed tight interaction of the NOX-E36 L-oligonucleotide with its target CCL2 is also reflected in the high binding affinity. By using SPR, a dissociation constant of 1.40±0.16 nM at physiological temperature of 37 °C was determined. This dissociation constant compares well with the affinities reported for other aptamers (reviewed in ref. 39) that were usually analysed at lower temperatures, such as room temperature. The high affinity of the NOX-E36 L-oligonucleotide is mainly facilitated by a slow off rate (Fig. 5), indicating a high complex stability, which is important for an efficient blocking of the CCL2 function.

For any pharmacological application of a drug substance, an understanding about its selectivity or specificity profile is useful if not mandatory. In binding studies employing SPR, we observed that the NOX-E36 L-oligonucleotide is able to recognize other chemokines that are related to CCL2, that is, CCL8, CCL11 and CCL13, but not CCL7. Compared with the binding kinetics of the L-aptamer and CCL2, association but also dissociation rates are accelerated with CCL8, CCL11 and CCL13, resulting in a slightly reduced affinity (Fig. 5). Now, the detailed analysis of the individual contacts between the NOX-E36 L-oligonucleotide and CCL2 provides a rationale for the observed selectivity profile of the L-aptamer: structural data suggest that the sequence deviations observed at positions 21 (serine to proline) and 63 (serine to phenylalanine or tyrosine) reduce the number of intermolecular hydrogen bonds that can be formed, and mutational studies confirmed that these sequence deviations indeed exhibit a negative effect on the affinity (Fig. 6), whereas other amino-acid substitutions R18K, V22K and H66Y did not cause a significant reduction in the affinity. The affinity of the L-aptamer for CCL8 and CCL11 is only slightly worse than for CCL2 (*K*_d_ increased by 2.4-fold) and the explanation apparently is that these two chemokines have a proline at position 21, which in the single point mutational analysis caused a sixfold reduction in the affinity. The S63Y mutation caused a stronger destabilization and consequently, the affinity of the L-aptamer for CCL13, which shows this amino-acid substitution, is weaker than for CCL8 and CCL11. The results highlight the importance of these interactions and it appears that the combination of both S21P and S63F substitutions occurring in CCL7 are important factors precluding its recognition by the L-aptamer. It is unclear, however, whether the cumulated effect of amino-acid substitutions S21P and S63F is sufficient to explain the non-binding of CCL7.

In a further analysis, we compared our structure with the previously published structures of two different antibody Fab fragments in complex with CCL2 (refs 40, 41; Supplementary Fig. 9). The first antibody, 11K2, binds to a relatively flat region on the CCL2 molecule, opposite to the receptor-binding site^41^. Although 11K2 neither binds to residues involved in dimer formation nor to those interacting with the receptor, the antibody was reported to block CCL2 action, probably by causing a steric hindrance of the CCL2–CCR2 interaction^41^.

In contrast, the antibody CNTO 888 (carlumab)^40^ binds to a part of CCL2 that is partially overlapping with the receptor interaction region. Twelve residues of CCL2 are involved in interaction, defined by a 4.0-Å distance cutoff: Arg18, Lys19, Ser21, Arg24 and Lys49 form hydrogen bonds and Ile20, Gln23, Thr45, Ile46, Val47, Ala48 and Ile51 are involved in van der Waals contacts^40^. Thus, the antibody epitope broadly overlaps with that of NOX-E36. The area covered on CCL2 is also similar for both substances (730 Å^2^ for CNTO888 and 748 Å^2^ for NOX-E36). The contacts made between the antibody and the CCL2 amino acids Ile46 and Val47 appear to be especially important for the high selectivity of CNTO888. Since Ile46 and Val47 are not conserved in related chemokines, the antibody consequently does not bind to CCL7, CCL8 or CCL13 (ref. 40) and cannot block CCR2 activation mediated by these chemokines. Clinical trials with CNTO888 led to disappointing results so far^42^,^43^, which was explained in part by a low affinity of the antibody under *in vivo* conditions^42^.

In conclusion, the combination of binding and structural data provides a detailed understanding of the interactions between the L-aptamer drug and its target L-CCL2 and insight into the drug's mode of action. The L-aptamer not only exhibits a high structural complementarity to its target but also efficiently blocks domains being essential for CCL2's *in vivo* functions, that is, the receptor-binding site and the GAG-binding region. On the other hand, the L-aptamer does not interfere with CCL2 dimerization and *vice versa*, dimerization does not interfere with L-aptamer binding. This is of importance because dimers or higher-order oligomers are the likely forms of CCL2 encountered *in vivo*. Thus, the molecular details support and are in line with the observations delineated from functional studies^14^,^34^. Together with the accompanying publication^15^, an interesting first insight into the recognition of natural L-proteins by mirror-image L-oligonucleotide aptamers is provided.

## Methods

### Expression and purification of recombinant CCL2

A cDNA sequence coding for mature human CCL2 (66 amino acids) was cloned into the pQE30Xa vector (Qiagen), thereby fusing the gene to an N-terminal His_6_-tag followed by a Factor Xa cleavage site, which allows to cleave off the His_6_-tag in order to obtain a protein with the natural N terminus. Cryocultures of *E.coli* BL21-pQE30Xa-CCL2 were incubated in 2YT medium overnight at 37 °C. This culture was diluted 1:25 and grown at 37 °C until the *A*_600_ reached 1.2. Expression was induced by addition of isopropyl-β-D-thiogalactoside to 0.5 mM and the cells were grown for 4 h before they were harvested. The cell pellet was resuspended in 50 mM NaH_2_PO_4_, 100 mM NaCl, pH 7.5 and homogenized before sonication at 4 °C (10 × 30 s). The resulting suspension was centrifuged at 19,000*g* for 30 min at 4 °C. The resulting pellets were resuspended in 100 NaH_2_PO_4_, 8 M urea, pH 8.0, followed by sonication (10 min, 4 °C) and centrifugation (19,000*g*, 4 °C, 30 min). The recombinant His-tag fusion protein was loaded and purified on HIS select gel matrix (Sigma Aldrich) in 100 mM NaH_2_PO_4_, 8 M Urea, pH 8.0, refolded on the resin by buffer exchange to 100 mM NaH_2_PO_4_, 50 mM NaCl, 10 mM glutathione, pH 8.0 and eluted with 200 mM imidazole in the latter buffer. The eluted and refolded protein was dialysed against 20 mM Tris-HCl pH 7.0, 50 mM NaCl overnight at 4 °C. The His_6_-tag was cleaved off using 20 U Factor Xa protease/mg protein (Qiagen). Factor Xa protease and CCL2 were separated by heparin affinity chromatography. The eluted CCL2 was dialysed against 20 m Tris-HCl pH 7.5, 100 mM NaCl overnight and the purity was analysed using SDS–PAGE.

Starting with the wild-type plasmid, mutants were generated using the QuikChange Lightning kit (Agilent). Introduction of mutations was verified by sequencing. For SPR measurements, wild-type and mutant CCL2 were expressed in *Escherichia coli* BL21 as described above and purified following a slightly varied protocol. Cell pellets were resuspended in 50 mM NaH_2_PO_4_, 100 mM NaCl, pH 7.5 incubated with lysozyme (200 μg ml^−1^) for 20 min at room temperature and homogenized in a French press. After centrifugation (19,000*g*, 4 °C, 30 min) the pellet was extracted with 100 mM NaH_2_PO_4_ pH 8.0, 8 M urea, 10 mM glutathione, 20 mM imidazole and centrifuged again. Using an ÄKTA Express instrument, the supernatant was then loaded on a HisTrap FF crude column (GE Healthcare) and the column was washed with the extraction buffer until the A_280_-signal reached a baseline. The proteins were refolded on the column by applying a 30-column volume gradient to 100 mM NaH_2_PO_4_ pH 8.0, 50 mM NaCl, 10 mM glutathione, 20 mM imidazole and eluted with 200 mM imidazole in that buffer. After concentration in an Amicon-ULTRA-4 centrifugal filter unit (3 kDa cutoff, Millipore), the buffer was exchanged to 20 mM HEPES, 100 mM NaCl, pH 7.4 using a PD-10 column (GE Healthcare). The His-tag was cleaved off by Factor Xa protease digestion (New England Biolabs). CCL2 proteins with native N terminus were then separated from protease and undigested protein by heparin affinity chromatography as described above. Purity was analysed using SDS–PAGE.

To establish the correct folding, the ability of CCL2 to activate the receptor CCR2 was controlled in chemotaxis assays with THP-1 cells (DSMZ no. ACC 16) as described earlier^12^.

### Oligonucleotide synthesis

The L-oligonucleotides with the sequence 5′- GCACGUCCCUCACCGGUGCAAGUGAAGCCGUGGCUCUGCG -3′ were synthesized using standard phosphoramidite chemistry at NOXXON Pharma AG (Berlin, Germany) essentially as described^44^. Regular L-phosphoramidites were purchased from ChemGenes (Wilmington, MA, USA). For the introduction of the seleno modification at position U31, the beta-L-5′-dimethoxytrityl-2′-deoxy-2′-methylseleno-3′-[(2-cyanoethyl)-(N,N-diisopropyl)]-uridine phosphoramidite (Rasayan Inc., Encinitas, CA, USA) was used; after coupling of methylseleno-uridine amidite, an additional cycle of treatment with 0.1 M dithiothreitol in ethanol/water 2:3 was carried out after the Cap/Ox/Cap treatment. Further working up and downstream processing remained unchanged.

### Complex formation

Recombinant human CCL2 and the L-oligonucleotide in binding buffer (20 mM HEPES pH 7.4, 100 mM NaCl, 5 mM KCl, 1 mM MgCl_2_ and 1 mM CaCl_2_) were mixed in equimolar ratio at 20 °C and incubated for 30 min. Specific complex formation was verified by dynamic light scattering and native polyacrylamide gel electrophoresis.

### SPR measurements

Binding affinities of the L-aptamer to human chemokines were determined on a Biacore 2000 instrument (BIACORE AB, Uppsala, Sweden). The chemokines were immobilized on a CM4 or CM5 sensor chips by an amine-coupling procedure on flow cells 2–4, whereas flow cell 1 served as dextran surface control. Hundred microlitres of a 1:1 mixture of 0.4 M (1-ethyl-3-(3-dimethylaminopropyl) carbodiimide in H_2_O) and 0.1 M N-hydroxysuccinimide in H_2_O were injected using the QUICKINJECT command at a flow of 10 μl min^−1^. Chemokines (data in Fig. 5: R&D Systems; data in Fig. 6: our own preparations) were dissolved in PBS pH 7.0 with 1% BSA to a concentration of 10 μM, diluted 1/100 in 10 mM sodium acetate pH 5.5 with 1 μM L-aptamer and subsequently 300–400 response units (RU) of the wild-type chemokines and CCL2 mutants were immobilized covalently on a CM4 sensor chips. Chemokines or CCL2 mutants that showed a low affinity or high binding rate constants, namely CCL13 and the CCL2 mutants R18A, K19A, R24A, S21P, S63F and S63Y, were immobilized covalently on a CM5 chip (4,000–5,500 RU) to allow more reliable fitting and data evaluation. The flow cells were blocked with an injection 70 μl of 1 M ethanolamine hydrochloride at a flow of 10 μl min^−1^.

Sensor chips were primed twice with degased physiological running buffer (20 mM Tris pH 7.4, 150 mM NaCl, 5 mM KCl, 1 mM MgCl_2_ and 1 mM CaCl_2_) and equilibrated at 50 μl min^−1^ until the baseline appeared stable. Before sample measurement, the chip underwent at least three injection and regeneration cycles.

The L-oligonucleotide was diluted in physiological running buffer and a concentration series (1,000; 500; 250; 125; 62.5; 31.3; 15.6; 7.8 (2 × ); 3.9; 1.95; 0.98 (2 × ); 0.48; 0.24; 0.12 nM) was injected, starting with the lowest concentration. In all experiments, the analysis was performed at 37 °C using the KINJECT command defining an association time of 240 and a dissociation time of 240 s at a flow of 30 μl min^−1^. The assay was double-referenced, whereas flow cell 1 served as (blocked) surface control (bulk contribution) and a series of buffer injections without analyte determined the bulk contribution of the buffer itself. At least one L-oligonucleotide concentration was injected a second time at the end of the experiment to monitor the regeneration efficiency and chip integrity during the experiments. Regeneration was performed by injecting 30 μl 5 M NaCl at a flow of 30 μl min^−1^. For kinetic evaluation of L-aptamer binding with high affinity to CCL2, CCL8, CCL11 and the CCL2 mutants R18K, V22K and H22Y with even very fast association rate constants (*k*_a_), only the concentration range of 1.95-0.98 (2 × ); 0.48; 0.24; 0.12; 0 nM was used for fitting the curve by a Langmuir 1:1 stoichiometric algorithm. Owing to the reduced affinity of the NOX-E36 oligonucleotide binding to CCL13 and the CCL2 mutants R18A, K19A, R24A, S21P, S63F and S63Y, a concentration range of 62.5; 31.3; 15.6; 7.8 (2 × ); 3.9; 1.95; 0.98 (2 × ); 0.48; 0.24; 0.12 nM was used for fitting the data. Data analysis and calculation of dissociation constants (*K*_d_) were performed with the BIAevaluation 3.1.1 software (BIACORE AB) with a refractive index correction set to zero and an initial mass transport coefficient *k*_t_ set to 1 × 10^7^ (RU M^−1^ s^−1^) for data fitting. The mean *K*_d_s were calculated from individually determined *K*_d_s and not from the mean *k*_a_ and *k*_d_.

### Crystallization

After optimization of complex formation conditions, initial crystals were obtained applying the Nucleic Acid Mini Screen (Hampton Research) for screening experiments. After thorough optimization of crystallization conditions, needle-shaped crystals (∼300 × 50 × 50 μm^3^) could be obtained by mixing 1 μl of the L-aptamer·CCL2 complex (in 20 mM HEPES pH 7.4, 100 mM NaCl, 5 mM KCl, 1 mM MgCl_2_, 1 mM CaCl_2_) with 1 μl of a solution containing 40 mM Na cacodylate pH 5.5, 12 mM spermine tetrahydrochloride, 40 mM LiCl, 80 mM SrCl_2_ and 20 mM MgCl_2_ and subsequent vapour-diffusion equilibration against 1 ml of reservoir (35% (v/v) 2-Methyl-2,4-pentanediol (MPD), 5 mM KCl, 1 mM MgCl_2_ and 1 mM CaCl_2_). Crystals suitable for data collection grew within 3 weeks at 4 °C.

### Data collection and processing

SAD diffraction data of the complex with Se-modified L-aptamer were collected at the PETRA III beamline P13 (EMBL Hamburg) at DESY (Hamburg, Germany) to 2.05 Å resolution with a Pilatus 6-M detector at a wavelength of 0.978 Å using a cryocooled crystal at 100 K (without further cryoprotection). Data processing was carried out with XDS^45^,^46^. The space group was assigned to P4_3_2_1_2 with unit cell dimensions of *a*=*b*=108.9 Å and *c*=34.8 Å.

### Structure determination and refinement

XDSCONV^45^,^46^, F2MTZ, and CAD (from the CCP4 suite^47^) were used to prepare the X-ray data for experimental phasing. The position of Se was determined with HySS^48^, experimental phasing followed by density modification was carried out with phenix.autosol^49^. The initial electron-density map after density modification was of sufficient quality to position and build one CCL2·L-aptamer complex present in the asymmetric unit. First, the RNA model with unusual ‘mirror-image' nucleotides was built manually applying Coot^23^. The required topology files for the nucleotides were calculated using the PRODRG2 server (http://davapc1.bioch.dundee.ac.uk/cgi-bin/prodrg). After fixing and refining the L-RNA position a part of protein close to the L-RNA was visible in the electron density map. Five amino-acid residues that have contacts with L-RNA were identified and a complete structure of the protein was superimposed to this fragment, using the coordinates of native CCL2 (ref. 18) deposited in the protein data bank (pdb code: 1DOL), allowing to complete the L-RNA·L-protein complex. REFMAC^50^,^51^ in combination with the inspection of the electron-density maps using the programme Coot^23^ was used to refine the model to 2.05 Å resolution with an *R* value of 20.0% and *R*_free_ of 25.0%. Ramachandran plot analysis showed that 98.5% of all residues were in the most favoured and the other 1.5% in additionally allowed conformations. Data collection and refinement statistics are summarized in Table 1. Data collection and refinement statistics are summarized in Table 1. In the final model 111 solvent water molecules could be identified.

### Identification and refinement of ions

One strontium ion, one sodium ion and five potassium ions were identified in the model at positions indicated by corresponding positive mFo–dFc electron densities. Ion positions were verified through careful inspection of coordination geometry^52^ using Coot^23^ and validation applying the CheckMyMetal server^24^. In order to verify this and to avoid model bias, phenix.refine^53^ was used for simulated annealing refinement (cooling down from 1,500 K to 300 K). The ions (Sr^2+^ and Rb^+^ in the case of the Sr^2+^ ion, Tl^+^, Cs^+^, Rb^+^, Mg^2+^ and Na^+^ in the case of Na^+^ and Tl^+^, Cs^+^, Rb^+^, Ca^2+^ and K^+^ in the case of K^+^) were re-introduced at the centre of the positive difference electron density peaks applying the programme Coot, followed by refinement with phenix.refine, applying manually added tight geometry restraints for the ion–oxygen distances (as reported in ref. 54) to assess the quality of ion placement. For Na^+^, Mg^2+^, K^+^ and Ca^2+^ distances were used as extracted from the Cambridge Structural Database^55^ in ref. 54, distances for Sr^2+^ (ref. 21), Cs^+^ (ref. 56), Tl^+^ (ref. 56) and Rb^+^ (ref. 22) were used as reported in the literature. The resulting ion–oxygen distances after refinement were inspected manually with Coot and cross-validated using the CheckMyMetal server^24^. In addition, anomalous difference Fourier electron density maps were calculated applying phenix.refine to further validate the ion positions. Theoretical f′′ values corresponding to an X-ray wavelength of 0.978 Å were calculated using the ‘Anomalous Scattering Coefficients' web tool (http://skuld.bmsc.washington.edu/scatter/).

### Figure generation and analysis of the structural model

Figures were generated using PyMol^57^ and the interaction between L-aptamer and CCL2 was analysed with PyMol, Chimera^58^, LigPlot+ (ref. 59) and PISA^25^. RNA characteristics were analysed using X3DNA (ref. 60). Note that because of the L-chirality of the aptamer, the output of the sugar puckering analysis has to be inverted with respect to endo/exo.

## Author contributions

S.K. and C.B. designed the experiment, K.B. cloned CCL2 and established expression and purification. S.F. expressed and purified CCL2. K.B. and J.A. produced CCL2 mutants. C.M. performed binding experiments. Complex formation and crystallization experiments were carried out by D.O. X-ray data collection was carried out by D.R., S.F. and D.O. X-ray data processing and experimental phasing of the X-ray data was carried out by D.O. Structural refinement was performed by A.G. and D.O. The experimental results were analysed by D.O., A.G., J.A. and C.B. D.O. and J.A. prepared figures. The manuscript was prepared by D.O., K.B., J.A., S.K. and C.B. with input from all authors.

## Additional information

**Accession codes:** Coordinates of the refined structural model and structure factors have been deposited to the Protein Data Bank (PDB) with the accession code 4R8I.

**How to cite this article:** Oberthür, D. *et al*. Crystal structure of a mirror-image L-RNA aptamer (Spiegelmer) in complex with the natural L-protein target CCL2. *Nat. Commun.* 6:6923 doi: 10.1038/ncomms7923 (2015).

## Supplementary Material

Supplementary Information Supplementary Figures 1-9 and Supplementary Tables 1-3

## Figures and Tables

**Table 1:** Data collection and refinement statistics.

*Data collection*	
Space group	*P4*_*3*_*2*_*1*_*2*
Unit-cell parameters a=b, c (Å)	108.91, 34.81
Resolution	77.0–2.05 (2.10–2.05)*
* R*_meas_ (%)	5.2 (32.3)
Average *I/*σ*(I)*	37.6 (6.8)
CC(1/2)	100.0 (95.5)
Completeness (%)	99.2 (95.1)
	
*Refinement*	
Resolution (Å)	77.0–2.05
No. of reflections	12,058 (771)
*R*_work_/*R*_free_	20.0/25.0
No. of atoms	
Protein	544
RNA	853
Ions	7
B factors	
Protein	42.98
RNA	26.39
Ions	26.43
R.m.s. deviations	
Bond lengths (Å)	0.011
Bond angle (°)	1.609

One crystal was used for the structure.

^*^Values in parentheses are for the highest resolution shell.
