# Supplementary material for: Crystal structure of a mirror-image L-RNA aptamer (Spiegelmer) in complex with the natural L-protein target CCL2
Source: Nat Commun. 2015 Apr 22;6:6923. doi: 10.1038/ncomms7923 (PMC4423205; doi:10.1038/ncomms7923)
Supplement: Supplementary Information — Supplementary Figures 1-9 and Supplementary Tables 1-3 [file ncomms7923-s1.pdf]

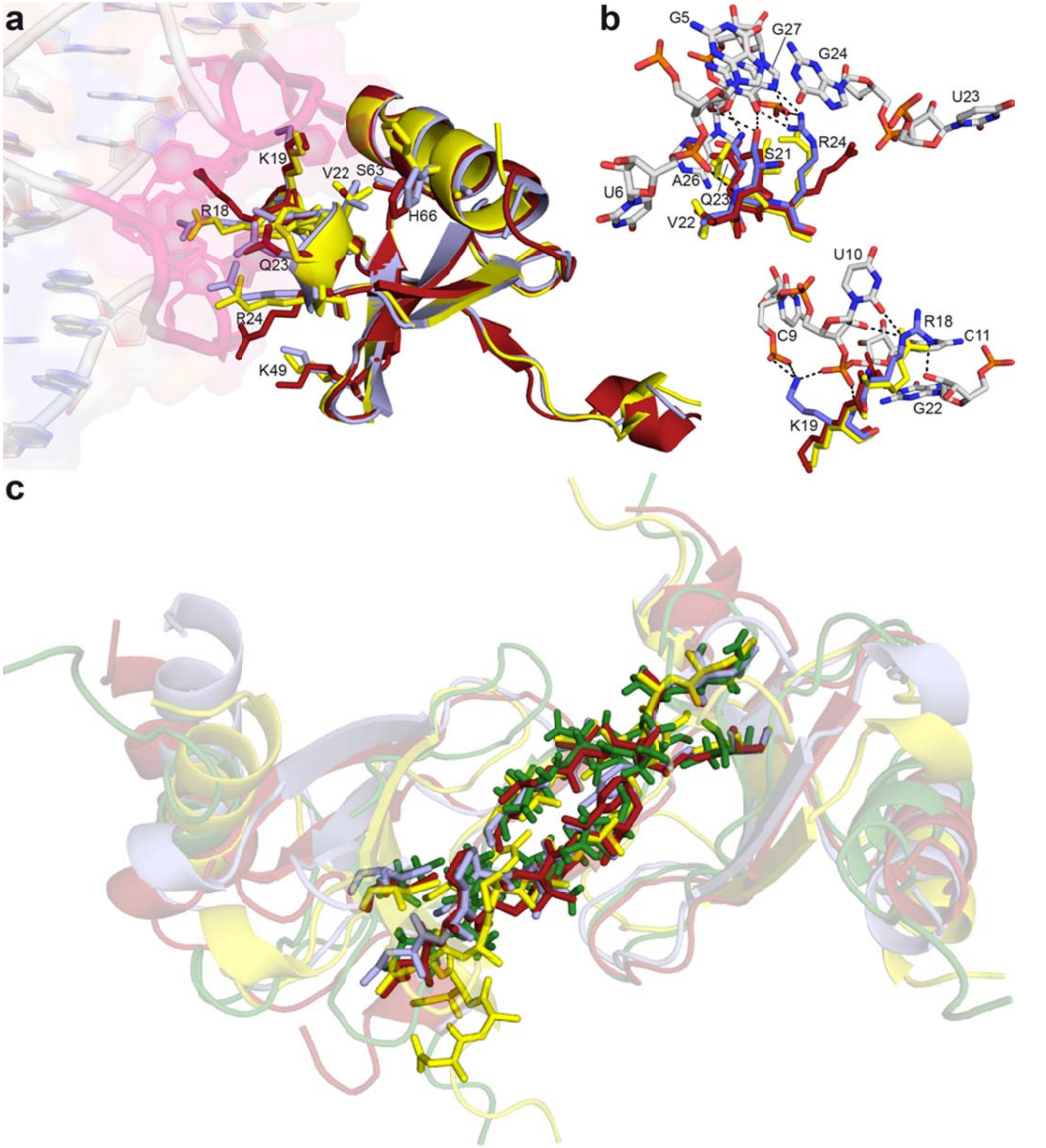

Supplementary Figure 1. Structural comparison of uncomplexed and L-aptamer bound CCL2. **(a)** Alignment of one monomer of uncomplexed CCL2 from crystal structures PDB 1DOL (red) and PDB 1DOK (yellow) with one monomer of CCL2 from our structure (blue) shown together with a semitransparent representation of the L-aptamer. Target-binding nucleotides are highlighted in magenta. The topology of all CCL2 structures is virtually identical, but different conformations of side chains within the epitope are observed for all three structures. With the exception of the Lys19, all side chains from our structure are seen in a similar conformation as observed in either of the other two structures. **(b)** Detailed view of the epitope (upper panel:

amino acids 20-24, lower panel: Arg18 and Lys19). **(c)** Detailed view and comparison of the CCL2 dimerization region from different structures. CCL2 in complex with the NOX-E36 L-aptamer is shown in blue. CCL2 is a dimer in the asymmetric unit for crystal structure PDB: 1DOK, shown in yellow and CCL2 is a monomer in the asymmetric unit for crystal structure PDB: 1DOL, shown in red, CCL2 derived from NMR studies, PDB: 1DOM, is shown in green. Interacting residues are displayed as sticks and the remaining part of the protein is shown as cartoon plot to highlight the overall orientation. For clarity reasons, the NOX-E36 L-aptamer was omitted in this figure.

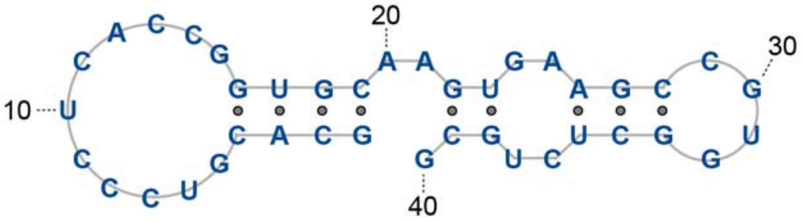

Supplementary Figure 2. Secondary structure of the NOX-E36 oligonucleotide as predicted by mfold.

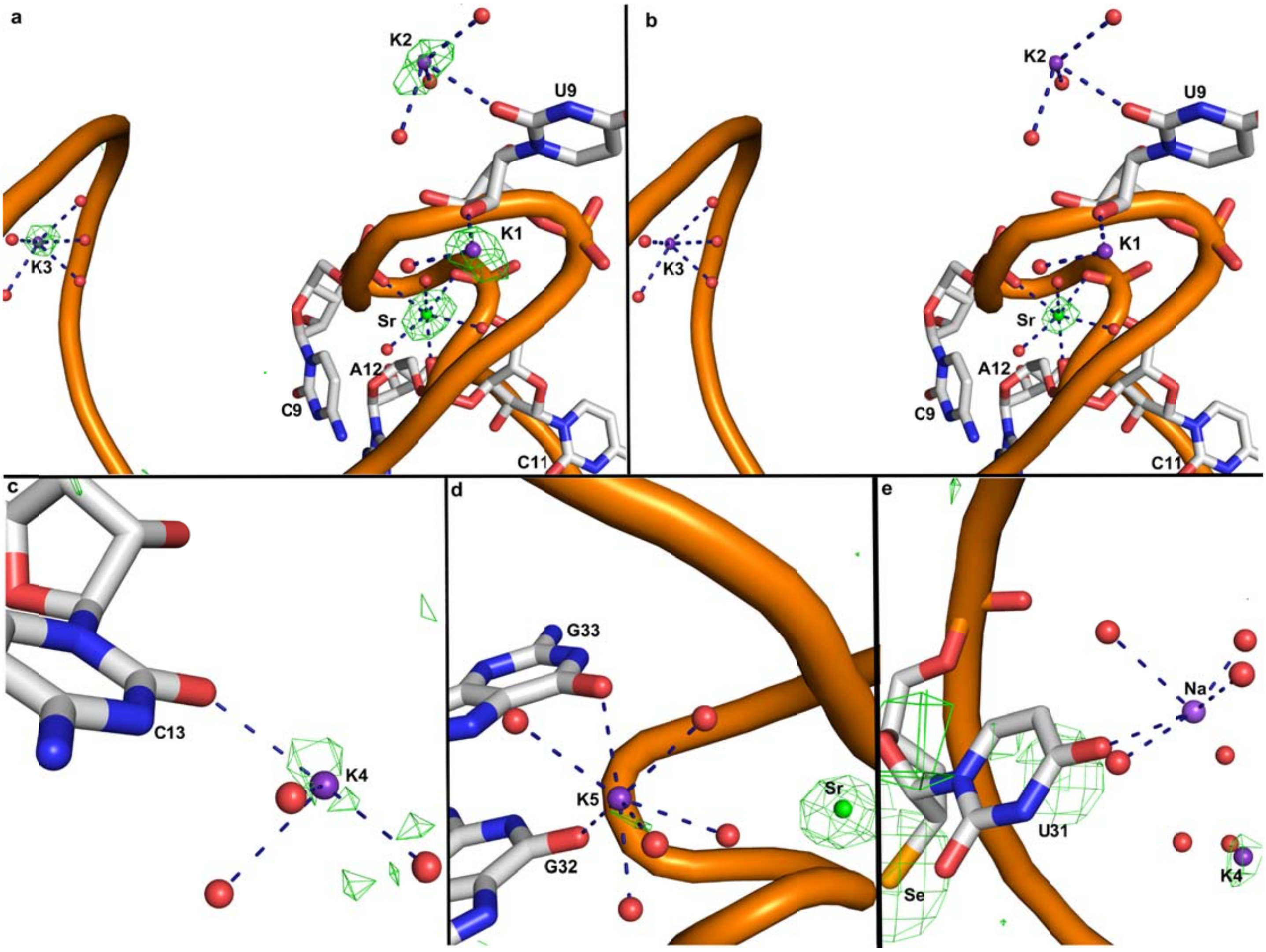

Supplementary Figure 3. (a) Anomalous difference electron density map (green mesh, contoured at 3σ) overlaid onto the final model. Sr^{2+}, three K^+ and their interaction partners are shown. **(b)** Same part of the model with the anomalous difference electron density map (green mesh) but contoured at 5σ, now only at the position of Sr^{2+} a electron density peak is visible. **(c)** K4 shows a weaker anomalous signal than K1-3 (as shown in (a), the anomalous difference electron density map (green mesh) is contoured at 2.6σ, since no peak at 3σ was visible. **(d)** Comparison of the anomalous map around K5 and Sr^{2+} contoured at 3σ. **(d)** No anomalous difference electron density is visible around Na^+ at 2.6σ, K4 is shown for comparative reasons. Na^+ interacts with 2'-methylseleno-uridine (U31), the strongest anomalous difference map peaks can be observed around the position of the Se atom.

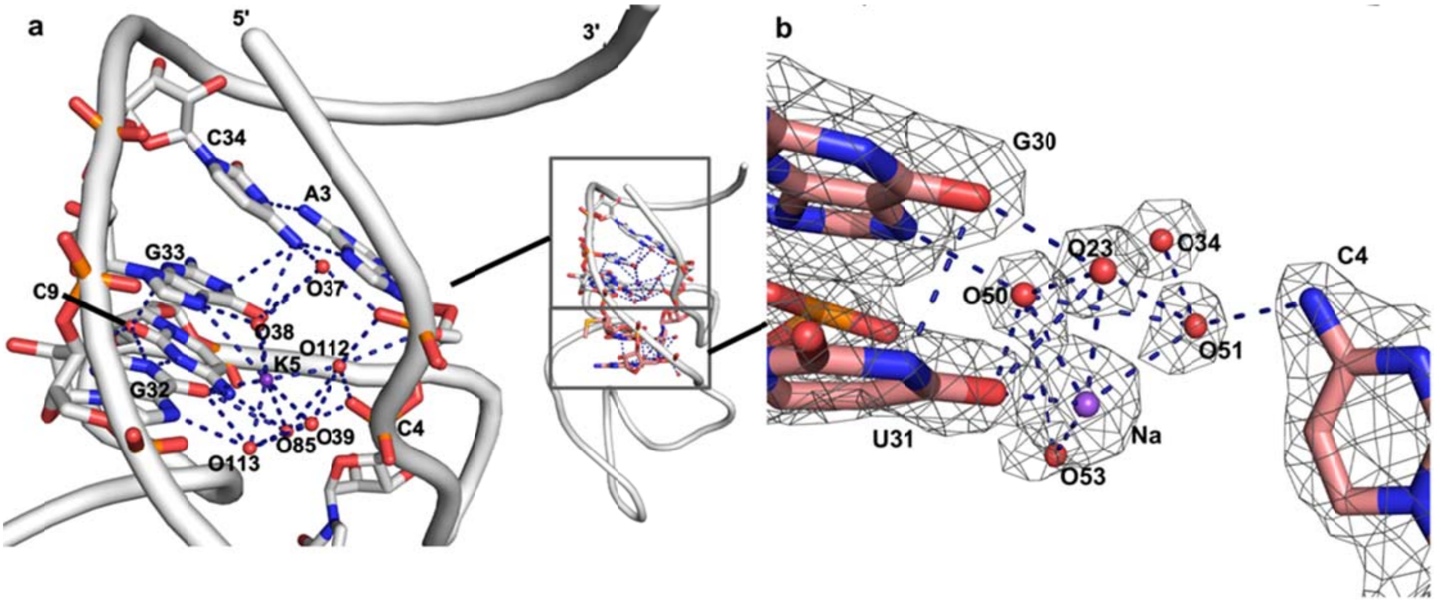

Supplementary Figure 4. (a) Detailed view of K5, its binding partners and the surrounding water network, interconnecting A3, C3, C9, G32, G33 and C34. The insert shows the position of this region relative to the overall structure of the aptamer. **(b)** Detailed view of the sodium ion and its chemical environment. Na^+ is connected to U31 and four water molecules. Through these water molecules, it is coordinated to C4 and G30.

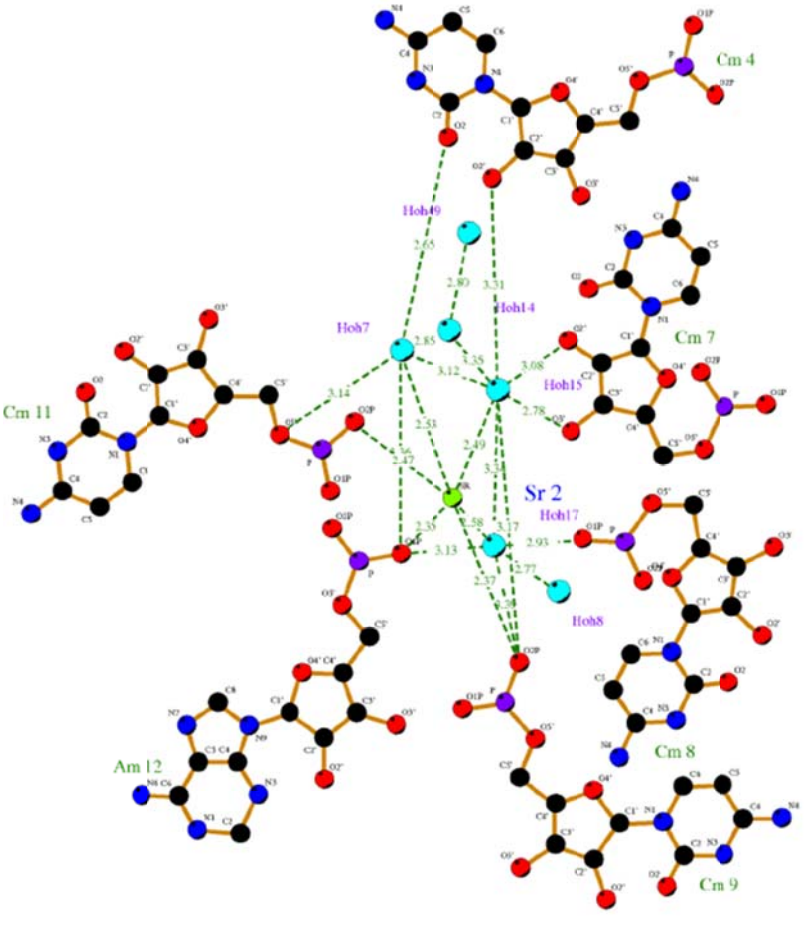

Supplementary Figure 5. Stabilization of the NOXE36 L-aptamer through Sr^{2+} and the associated water network. A 2D plot of the strontium ion (green) interacting with surrounding water molecules (cyan) and the L-oligonucleotide residues shows the stabilizing effect of the bivalent cation. Sr^{2+} forms direct salt bridges with the phosphate backbone oxygens of residues C9, C11 and A12. Moreover, it is directly coordinated by three water molecules and through this water network, it is connected to the phosphate of C8, O2’ and O3’ of C7 and to O2 and O2’ of C4. C7-C11 are directly involved in target binding.

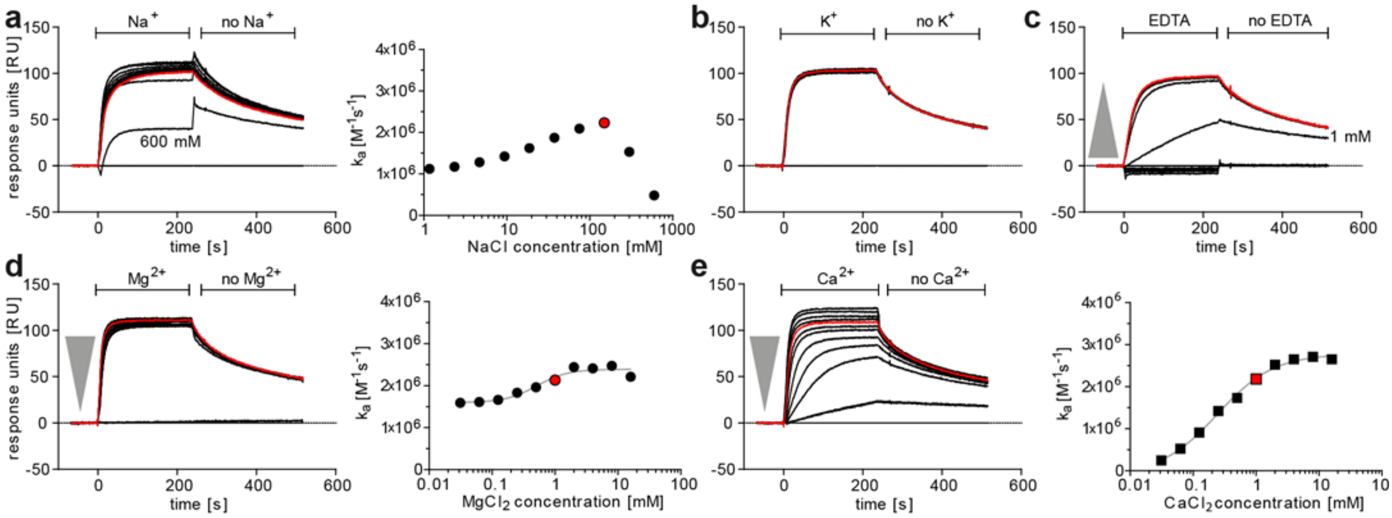

Supplementary Figure 6. Ion dependence of NOX-E36 binding to CCL2. All SPR measurements were performed in physiological running buffer, whereby one ingredient was permutated. In all graphs, red lines or dots mark unaltered physiological conditions. **(a)** Sodium was permutated from 2 mM to 600 mM. The association rate peaks at physiological concentrations. **(b)** Potassium was permutated. No concentration dependent effect was observed. **(c)** Bivalent ions were depleted with increasing amounts of EDTA. No binding was observed with EDTA concentrations exceeding 1 mM, thus bivalent ions are essential for binding. **(d)** Magnesium was permutated from 20 µM to 20 mM, revealing a minor positive correlation of magnesium concentration and association rate. Magnesium does not appear to be essential for binding. **(e)** Calcium was permutated from 20 µM to 20 mM, exhibiting a strong positive correlation of calcium concentration and association rate. Calcium appears to be essential for binding.

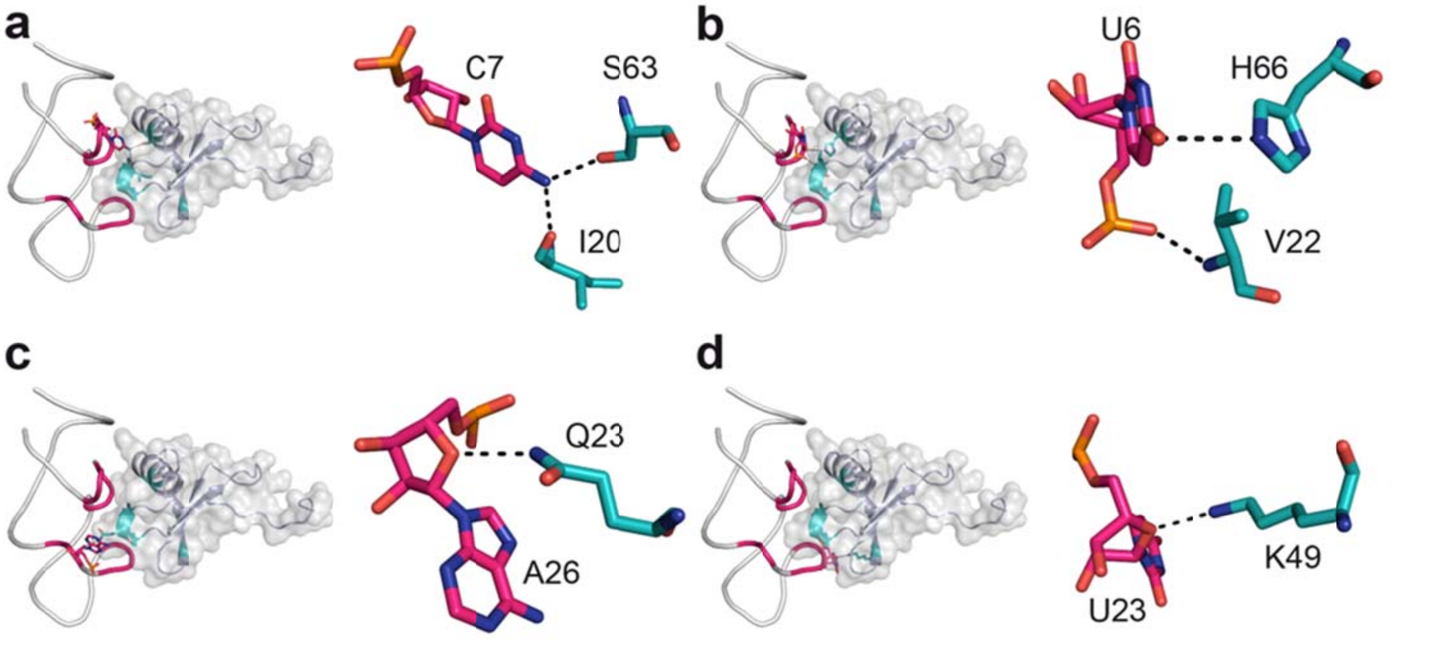

Supplementary Figure 7. Interactions between L-aptamer and CCL2 (continued from Fig. 3). **(a)** Both the main chain carbonyl of Ile20 and the side chain hydroxyl group of Ser63 are forming in hydrogen bonds with the N4 of nucleotide C7. **(b)** A phosphate oxygen of nucleotide U6 binds to the main chain nitrogen of Val22 and the τ-nitrogen of His66 forms a hydrogen bond to the O4 of the same nucleotide. **(c)** The side chain nitrogen of Gln23 makes a hydrogen bond to the 4'O of nucleotide A26. **(d)** The ε-nitrogen of Lys49 binds to the 4'O of nucleotide U23.

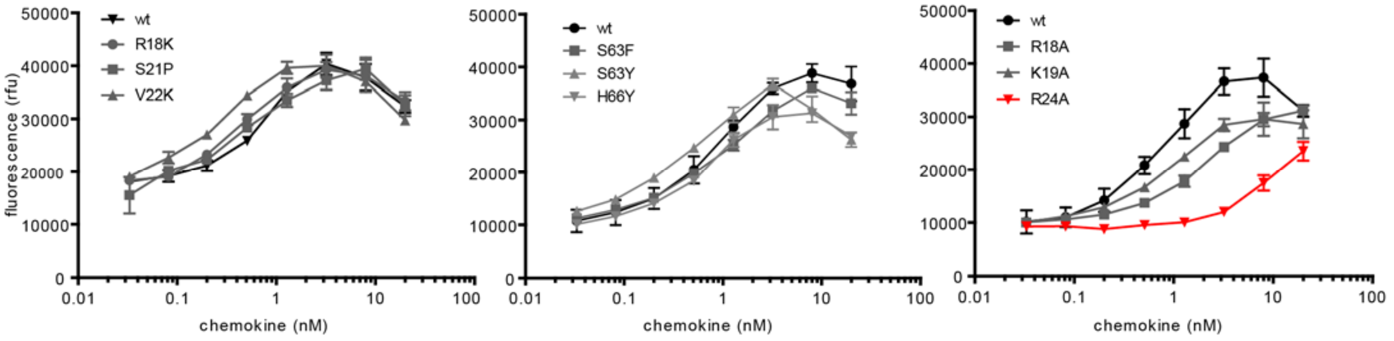

Supplementary Figure 8. Chemotaxis assays with recombinant wild type and mutant CCL2. To control whether the protein preparations contain CCL2 in its active conformation, their ability to trigger chemotaxis was controlled and all preparations passed the test. CCL2 mutant R24A is known for defective receptor binding, in which the native amino acid Arg24 is directly involved. This mutant consequently shows a reduced potential to trigger chemotaxis. Data points represent means ±S.E. of triplicates from one experiment each.

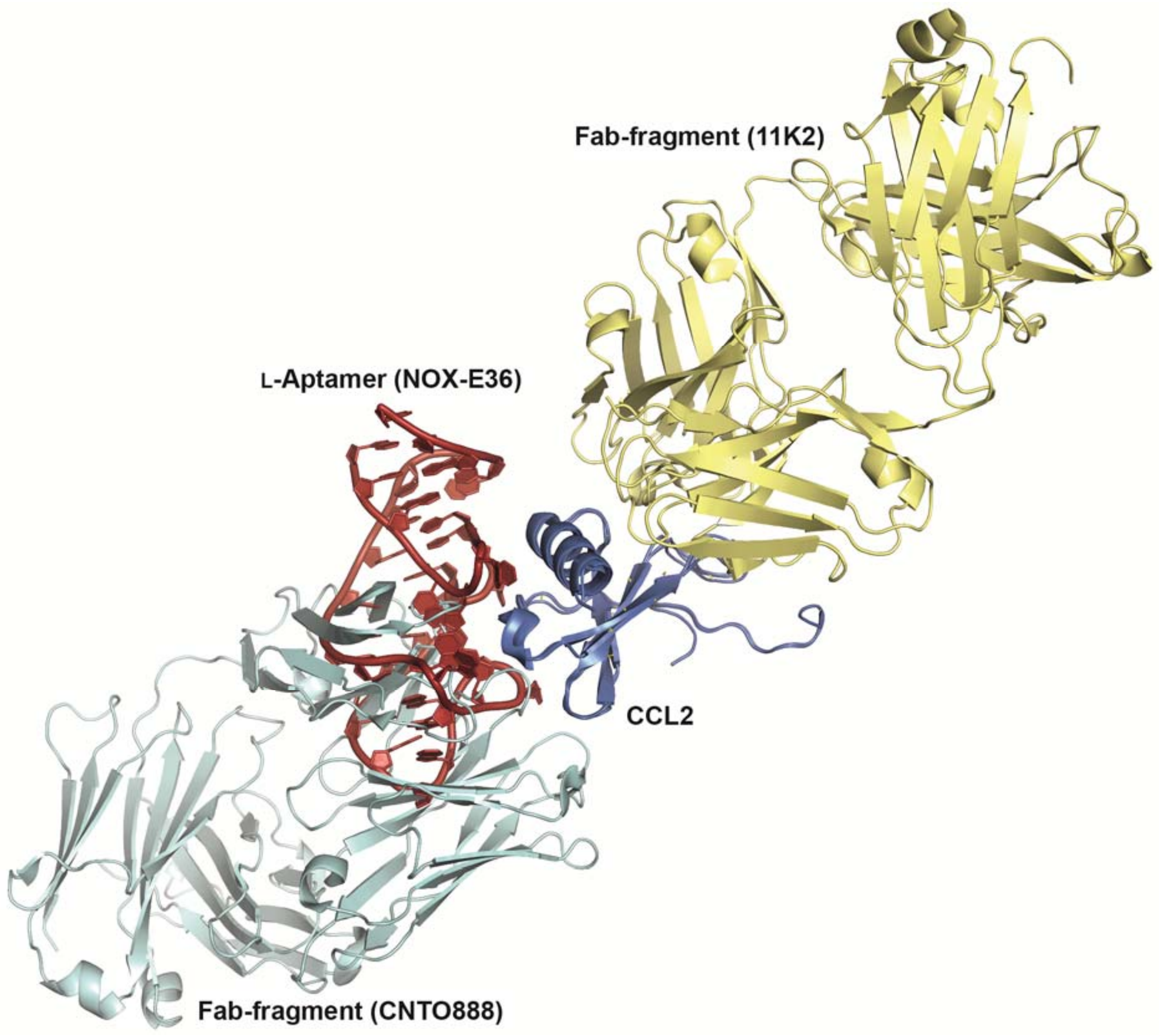

Supplementary Figure 9. CCL2/MCP-1 recognition by antibodies and the L-aptamer. The superimposition of the crystal structures of CCL2 (blue) in complex with the NOX-E36 L-aptamer (red) and Fab-fragments of antibodies 11K2 (PDB: 2BDN, yellow) and CNTO888 (PDB: 4DN4, cyan) shows that the two antibodies bind to opposite sides of CCL2 (blue). The epitope of the L-aptamer overlaps with the epitope of the antibody CNTO888, but additionally binds to functionally relevant amino acid residues located in the C-terminal α-helix.

Supplementary Table 1. Results of superimposing CCL2 structural models deposited in the PDB to CCL2 in complex with NOX-E36 and to each other using all atoms.

Reference Molecule	Moving Molecule	r.m.s.d. (Å)
CCL2 in complex with NOX-E36	CCL2 (1DOL)	0.60
CCL2 in complex with NOX-E36	CCL2 (1DOK, Chain A)	0.41
CCL2 in complex with NOX-E36	CCL2 (1DOM, Chain A, NMR structure)	1.26
CCL2 (1DOL)	CCL2 (1DOK, Chain A)	0.50
CCL2 (1DOL)	CCL2 (1DOM, Chain A, NMR structure)	1.38
CCL2 (1DOK, Chain A)	CCL2 (1DOM, Chain A, NMR structure)	1.21

Supplementary Table 2. NOX-E36 oligonucleotide base pairings

Watson-Crick	Hoogsteen	Non-Canonical
G1-C39		
C2-G38		
A3-U37	A3-C34	
C4-G22		
		G5-G24
C8-G33		
C9-G32		
C15-G30		
G15-C29		
G16-C28		
	U17-A21	
		G18-A25

Supplementary Table 3, Refinement of ion-oxygen distances.

**Average ion - oxygen distances (Å)**	**Sr**		**K1**	**K2**	**K3**	**K4**	**K5**		**Na**
In the final model	2.47±0.09	In the final model	2.60±0.11	2.59±0.08	2.45±0.10	2.59±0.09	2.63±0.22	In the final model	2.51±0.23
After Sr^{2+} removal followed by simulated annealing refinement	2.69±0.05	After ion removal followed by simulated annealing	2.69±0.09	2.75±0.25	2.42±0.23	2.53±0.33	2.60±0.45	After ion removal followed by simulated annealing	2.67±0.27
After adding back Sr^{2+} and imposing tight geometry restraints on the Sr^{2+}-O distances	2.55±0.08	After adding back K^+ and refinement with tight geometry restraints on metal-oxygen distances	2.65±0.02	2.72±0.03	2.58±0.10	2.69±0.08	2.68±0.11	After adding back Na^+ and refinement with tight geometry restraints on metal-oxygen distances	2.50±0.11
After adding Rb^+ and imposing tight geometry restraints on the Rb^+-O distances	2.64±0.20	After adding back Ca^{2+} and refinement with tight geometry restraints on metal-oxygen distances	2.53±0.05	2.56±0.06	2.40±0.01	2.50±0.12	2.56±0.17	After adding back Mg^{2+} and refinement with tight geometry restraints on metal-oxygen distances	2.42±0.14
		After adding back Rb^+ and refinement with tight geometry restraints on metal-oxygen distances	2.72±0.06	2.79±0.03	2.66±0.06	2.81±0.12	2.77±0.10	After adding back Rb^+ and refinement with tight geometry restraints on metal-oxygen distances	2.86±0.09
		After adding back Cs^+ and refinement with tight geometry restraints on metal-oxygen distances	2.89±0.05	2.86±0.06	2.77±0.14	3.00±0.08	2.86±0.12	After adding back Cs^+ and refinement with tight geometry restraints on metal-oxygen distances	2.96±0.19
		After adding back Tl^+ and refinement with tight geometry restraints on metal-oxygen distances	2.89±0.07	2.88±0.05	2.81±0.30	2.87±0.13	2.89±0.08	After adding back Tl^+ and refinement with tight geometry restraints on metal-oxygen distances	2.97±0.12

Distances used for geometry restraints: Na^+--O = 2.42 Å, Mg^{2+}--O = 2.08 Å, Ca^{2+}--O = 2.43 Å , K^+--O = 2.81 Å, Sr^{2+}--O = 2.62 Å, Rb^+--O = 2.98 Å, Os^+--O = 3.05 Å and Tl^+--O = 2.80 Å.
